# Surrendered and Stray Dogs in Australia—Estimation of Numbers Entering Municipal Pounds, Shelters and Rescue Groups and Their Outcomes

**DOI:** 10.3390/ani7070050

**Published:** 2017-07-12

**Authors:** Diana Chua, Jacquie Rand, John Morton

**Affiliations:** 1 School of Veterinary Science, University of Queensland, Gatton, QLD 4343, Australia; j.rand@uq.edu.au (J.R.); john.morton@optusnet.com.au (J.M.); 2 Australian Pet Welfare Foundation, Kenmore, QLD 4069, Australia; 3 Jemora Pty Ltd., PO Box 2277, Geelong, VIC 3220, Australia

**Keywords:** surrendered, stray, dogs, animal shelter, municipal pound, euthanasia, Australia, rehome, reclaim, transfers, rescue

## Abstract

**Simple Summary:**

Analyses of comprehensive and accurate dog intake and outcome data in municipal pounds and shelters across states in Australia would provide an in-depth understanding of the surrendered and stray dog issue as well as facilitate effective evaluation of existing management strategies. Currently, there is a lack of comprehensive and reliable data at the federal, state and local government levels across public and private agencies. In this study, we developed a methodology to estimate the annual numbers of dog admissions in Australia, and to describe their outcomes. In 2012–2013, there were an estimated 9.3 dog admissions per 1000 residents (211,655 dog admissions). Of these admissions, 4.4 per 1000 residents were reclaimed (101,037 reclaimed), 2.9 per 1000 residents were rehomed (66,443 rehomed) and 1.9 per 1000 residents were euthanized (43,900 euthanized). An ongoing standardized monitoring system would enable Australia to evaluate management strategies to reduce numbers of dogs admitted and euthanized, and to benchmark its unwanted dog management policies and performance against comparable countries.

**Abstract:**

There is no national system for monitoring numbers of dogs entering municipal council pounds and shelters in Australia, or their outcomes. This limits understanding of the surrendered and stray dog issue, and prevents the evaluation of management strategies. We aimed to estimate these in 2012–2013. Dog intake and outcome data were collected for municipal councils and animal welfare organizations using annual reports, publications, primary peer-reviewed journal articles, websites and direct correspondence. More comprehensive data were obtained for New South Wales, Victoria, South Australia and Australian Capital Territory, whereas it was necessary to impute some or all data for Western Australia, Northern Territory, Queensland and Tasmania, as data were incomplete/unavailable. A refined methodology was developed to address the numerous limitations of the available data. An estimated national total of 211,655 dog admissions (9.3 admissions/1000 residents) occurred in 2012–2013. Of these admissions, the numbers where the dog was reclaimed, rehomed or euthanized were estimated as 4.4, 2.9 and 1.9/1000 residents, respectively. Differences in outcomes were evident between states, and between municipal councils, welfare organizations and rescue groups. This study emphasizes the need for an ongoing standardized monitoring system with appropriate data routinely collected from all municipal councils, animal welfare organizations and rescue groups in Australia. Such a system would only require data that are easily collected by all relevant organizations and could be implemented at relatively low cost. This could facilitate ongoing evaluation of the magnitude of the surrendered and stray dog problem, and allow assessment of strategies aiming to reduce numbers of admissions and euthanasia.

## 1. Introduction

Surrendered and stray dogs pose a substantial problem in most Western countries. Large numbers of dogs enter municipal council pounds and animal welfare shelters because of changes in owner circumstances, such as personal crises, lack of “pet-friendly” accommodation, travelling, changes in relationships or income, and as a result of unplanned canine pregnancies [1]. Escaped or wandering dogs are also a major contributor to dog intake. The management of these animals by public authorities and animal welfare agencies has become increasingly contentious with euthanasia being employed as a population control strategy [2]. 

The American Society for Prevention of Cruelty to Animals estimated that 3.3 million dogs (10.2/1000 residents) are admitted to shelters operated by welfare agencies and municipal governments, and 670,000 dogs (2.1/1000 residents) are euthanized annually, although the method used for generating these estimates was not reported [3]. In a 2010 study in the United Kingdom, 121,693 dogs (1.9 per 1000 residents) were admitted to animal welfare organizations [4], and 10.4% were euthanized [5]. In Australia, comprehensive data are not available, although Death Row Pets estimated 250,000 healthy but unwanted dogs and cats are killed in Australian municipal pounds each year. However, they did not report numbers separately for dogs and cats [6], and it is unknown what methods were used to estimate this. 

The management of stray and surrendered dogs and cats has a high socio-economic cost. In the United Kingdom, expenditure by animal welfare organizations was £340 million in 2010 [7], and £57.5 million was spent by local authorities in 2011 [8]. In Australia in 2004, the cost of managing stray and surrendered animals was estimated to be $263 million annually ($83 million in municipal council pounds and $180 million in welfare shelters) [9,10]. Furthermore, using euthanasia as a population control strategy has a human cost, creating a “caring-killing paradox” [11,12], where shelter workers struggle constantly with the moral dilemma of their job. This can result in “negative coping”, such as substance abuse or heighten suicidal tendencies [12,13]. Staff turnover increases as euthanasia percentages increase [14]. These issues have since fueled critical appraisal of the management strategies in municipal pounds and shelters in many countries. 

In Australia, surrendered and stray dogs are accepted and managed by municipal councils (local government), animal welfare organizations, animal rescue groups and other charities. Animal welfare organizations and animal rescue groups are non-government organizations. Shelters operated by animal welfare organizations must comply with specific legislative requirements, and larger scale organizations typically operate at state or national level e.g., the Royal Society for the Prevention of Cruelty to Animals (RSPCA) [15], Animal Welfare League (AWL) [16] and the Lost Dogs Home (LDH) [17]. Animal rescue groups are local community volunteer organizations, usually operating on a foster-care model.

Currently in Australia, there is no national or state-based system for ongoing monitoring of numbers of admissions of dogs into pounds and shelters and their outcomes. This lack of meaningful information makes it impossible to evaluate the effectiveness of existing strategies in addressing these issues [18]. Access to accurate, comprehensive statistics and performance indicators would assist in identification of the strengths and weaknesses of existing management strategies of stray and surrendered dogs [19] and facilitate effective allocation of resources in government and animal welfare organizations [20]. Lack of such data forms a significant barrier to improving outcomes for dogs admitted to shelters and pounds in Australia. The aims of this study were to determine the availability and quality of data sources, and to develop the methodology to estimate the numbers of dogs entering Australian municipal council pounds, animal welfare organizations and animal rescue groups for 2012–2013, and describe their outcomes on a national and state level, and by type of organization.

## 2. Materials and Methods

### 2.1. Organizations

Municipal councils are local governments which handle community needs including animal control. In every state, these were identified through relevant state government websites and were contacted by emails or phone calls to determine if they operated a municipal council pound (animal control facility), and to request admission and outcome data for dogs in 2012–2013. We utilized local knowledge and the internet to identify the animal welfare organizations in each state that operated animal shelters with estimated intakes of 1000 or more dogs, and obtained data from their website where available, or through email and phone requests. Small shelters that were independent of larger welfare organizations, and thought to have annual intakes of less than 1000 dogs were not contacted because of the difficulty identifying and contacting them. Rescue group data in New South Wales for 2012–2013 were obtained from the New South Wales Office of Local Government. Animal Management in Rural and Remote Indigenous Communities (AMRRIC) manage dog populations in indigenous communities in the Northern Territory through desexing and health programs, but no data from AMRRIC were included because they do not operate any physical pounds or shelters.

### 2.2. Definitions of Intake and Outcomes

Intake was defined as the number of admissions in the year, where a dog entering a council pound, a shelter managed by an animal welfare organization, or the care of an animal rescue group constituted one admission. After entry, some dogs were transferred to another organization (municipal council, animal welfare organization or animal rescue group). Thus, the intake for a particular organization could consist of (a) dogs brought in by the general public or stray dogs captured by authorities, etc. and (b) dogs transferred in from another organization. The five possible outcomes of an admission were: the dog was reclaimed by their owner, rehomed (i.e., allocated to a new owner), experienced an “other” outcome (died in the shelter, stolen, lost etc.), was transferred out, or was euthanized. At state/territory and national levels, the definition of an admission did not include dogs transferred between organizations. Any particular dog could contribute to multiple admissions in the same year.

### 2.3. Data Collection

Data were collected in 2014–2015. Table A1 summarizes the sources of data. Data for municipal councils and animal welfare organizations that operated shelters that received surrendered and stray dogs were obtained from their websites, where available. When unavailable, data were solicited by direct communication with the organization by email and/or telephone using contact information from websites. Contacted organizations were assured that their anonymity would be preserved, and that data, which were not publically available, would not be released in a way that allowed identification of the individual organization without written permission. 

Emails requesting data were sent to all municipal councils in Queensland, Northern Territory, Western Australia and Tasmania and followed up by additional emails and/or telephone to non-responders. Municipal council data for municipal councils in the other states and territories were obtained without direct contact. For New South Wales, aggregated council data had been collated by the New South Wales Department of Local Government [21], and for South Australia, the Dog and Cat Management Board provided pooled data for both municipal councils and animal welfare organizations. For the Australian Capital Territory, Domestic Animal Services, a section within the territory’s Department of Local Government, provided data for its only state-run council pound. For Victoria, data were available for most municipal councils on their websites; some councils were also contacted by phone for information.

While we sought data for the 2012–2013 financial year in Australia (i.e., 1 July 2012 to 30 June 2013), for a minority of organizations, data were available only for 2011–2012 or for the calendar year 2012, or an earlier year. These data were still utilized in the study, based on the assumption that it was more accurate to include their data than to exclude it. 

### 2.4. Estimation of Unavailable Council Data

For each municipal council, we attempted to ascertain whether the council managed their pounds themselves or whether other councils, animal welfare organizations or animal rescue groups managed the council’s pounds. If the former, we also attempted to ascertain whether the council rehomed unclaimed dogs and/or transferred unclaimed dogs to other councils, welfare organizations or rescue groups. 

Where data were available for a council, and an animal welfare organization managed that council’s pounds, we attempted to ascertain whether the welfare organization excluded council data from their statistics. If this was known to be the case, then the council pound data were added to the welfare agency’s statistics. Otherwise, council data were assumed to have been included in the welfare organization’s reported data. This was also assumed where no data were available for a council and the council had a contractual obligation with a welfare organization (e.g., 84Y agreement in Victoria [22]). In the case of South Burnett Council (Queensland), the council operated all of its pounds with one exception, Kingaroy, which was operated by the RSPCA; for calculation purposes, all pounds in South Burnett were assumed to have been operated by the council. For Ipswich council, data were included in AWL QLD’s shelter data, and hence were excluded. 

**Figure A1:**
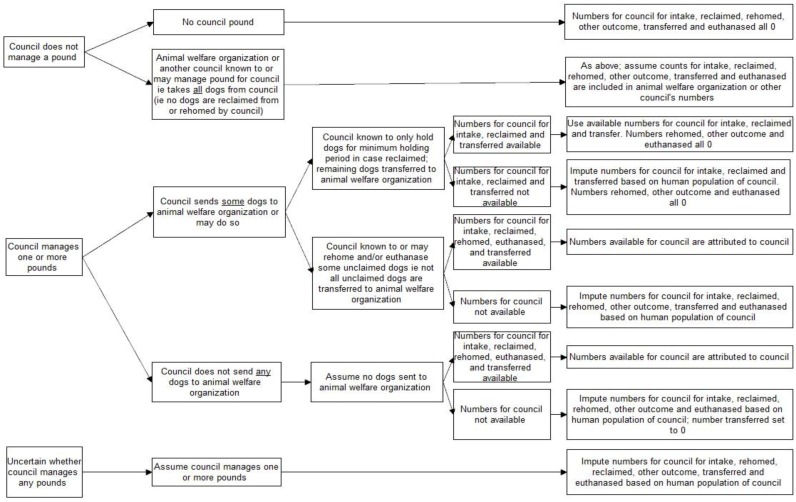
Algorithm of assumptions used to estimate numbers for municipal councils with incomplete or no data.

Not all municipal councils provided complete data and some councils provided no data. Therefore, for municipal councils with incomplete or no data, we imputed the missing numbers based on pooled data from all other councils in that same state or territory whose data were available and the council’s human population. For municipal councils whose data were available, the ratio of the pooled intake to the human population sizes for those councils pooled was calculated. For each municipal council where the intake number was not available, this number was imputed by multiplying the council’s human population size by this ratio. The same process was used to impute numbers of dogs reclaimed, rehomed, transferred, euthanized or had another outcome (died, escaped etc.). Human population numbers were obtained from the Australian Bureau of Statistics [23] and estimated resident populations by local government area (i.e., by council area [24]) as on 30 June 2012 were used. The term “in care” referred to dogs at the start and end of the year that were in the pound or shelter, or in foster care, external to the admitting facility but still under the care and responsibility of the pound or shelter. Numbers of dogs in the care of the council at the beginning and end of the year were not available for any council, so when making these estimates within each council, we assumed that numbers in care were the same at the beginning and end of the year. The algorithm for determining these numbers is shown in Figure A1.

Pooled data were provided by the South Australian Dog and Cat Management Board for municipal councils and animal welfare organizations for numbers of dogs admitted and reclaimed, but numbers transferred, rehomed and euthanized for councils were not included in the pooled data. Therefore, for South Australian councils, we calculated intake and the number reclaimed from municipal councils, using the data provided minus numbers for each of the two animal welfare organizations operating in that state (RSPCA data from website, AWL provided data on request). We then estimated the numbers rehomed, transferred, having an “other” outcome, and euthanized based on the percentages of these outcomes for unclaimed dogs pooled for municipal councils in other states and territories where raw data were available. We multiplied the total number unclaimed for South Australian councils pooled (i.e., number admitted minus number reclaimed) by each of these percentages. We assumed there were no specific state-wide factors affecting outcomes for animals in municipal councils in South Australia that were different from other states, because RSPCA outcomes were similar to the mean outcomes for the rest of Australia.

### 2.5. Numbers for Animal Rescue Groups

Animal rescue groups were not required by authorities to report data centrally in any state or territory other than New South Wales. Due to the large number of animal rescue groups and difficulty in identifying all such groups, we did not contact these. We instead estimated numbers for animal rescue groups pooled within each state and territory based on actual data routinely collected in New South Wales. In New South Wales, animal rescue groups that were registered with government were exempt from having to pay council dog registration (dog license) fees for the first 12 months that an individual animal was in their care under a 16d exemption. In return, registered groups were requested to submit summarized annual reports to the state’s Office of Local Government. Submitted data for 2012–2013 were provided by an animal advocacy group (Justice4Max) [25], which obtained them via an informal Freedom of Information request (GIPA). However, many groups reported numbers for dogs and cats pooled and not separately for each species. Based on numbers from groups where the group’s focus (dogs, cats or both) was indicated in their name, or where this was known, we estimated that three-quarters of the 7671 intake to these animal rescue groups in New South Wales were dogs (data not shown). Based on this knowledge of animal rescue groups and data from New South Wales showing 2% of dogs were euthanized, for each state and territory, we further assumed that no dogs entering animal rescue groups were reclaimed or transferred, and 98% were rehomed (pers comm. Geoff Davidson, Max4Justice/New South Wales).

We also estimated that the reported numbers represented approximately two-thirds of animals admitted to animal rescue groups, because only 52 (60%) of the 86 registered animal rescue groups had submitted data, other animal rescue groups were not registered, and it was likely that the animal rescue groups that had submitted data were those with larger intakes. Accordingly, we estimated the intake to animal rescue groups in New South Wales in 2012–2013 as 8630 (7671 × 3/4 then divided by 2/3). For each state and territory, we assumed intakes to animal rescue groups were the same per 1000 human population as for New South Wales because we had no evidence of specific state-based factors that made another assumption more correct, and omitting these data would have led to a larger error when estimating numbers of dogs rehomed. Human population sizes for each state and territory were obtained as described above [23]. 

### 2.6. Numbers for Western Australia

We initially collected and imputed Western Australian council data as described above for other states and territories. However we could only obtain actual data for 9 (6%) of the 141 Western Australian municipal councils, and the estimated intake to council pounds (12,720) based on human population sizes constituted a large proportion (58%) of the calculated state total admission. Furthermore, in Western Australia, numerous municipal councils shared pounds, dogs were often transferred to other councils, and it was difficult to obtain details about these for many councils, making it challenging to identify which councils had pounds, and which council pounds were operated by other organizations. This raised the possibility of double counting some intakes and failing to count others. Accordingly, for Western Australia, we estimated numbers for municipal councils, animal welfare organizations and animal rescue groups pooled based on the human population of the state, assuming numbers would be the same per 1000 human population as for all other states and territories pooled. We then subtracted numbers from the one known welfare organization in Western Australia (RSPCA), and estimated numbers for rescue groups as described above; the remainder constituted the imputed numbers for municipal councils pooled.

### 2.7. Calculation of Total Numbers of Admissions and Outcome Statistics

Numbers of dogs in care at the start and end of the year were obtained where available. For the RSPCA, the recorded number of admissions for the year included numbers in care at the start of the year, so we deducted these to obtain intakes during the year. 

After accounting for any differences between the numbers in care at the start and end of the year, for some organizations, the reported intake was less than the total of the numbers of admissions ending in each of the 5 possible outcomes. For these, we assumed numbers for each outcome and numbers in care were correct, and intake numbers understated, and added the discrepancy to the state’s or territory’s recorded total intake. Similarly, for other organizations, after accounting for any differences between the numbers in care at the start and end of the year, the intake number was more than the total of the numbers of admissions having each outcome. For these, we assumed numbers for each outcome and numbers in care were correct and intake numbers overstated, and so deducted the discrepancy from the state’s or territory’s recorded total intake. The net values of these discrepancies (ignoring the direction of the discrepancy) were 0.0% to 5.2% of the state’s or territory’s recorded total intake except for Northern Territory (6.9% or 268) and South Australia (17.0% or 3190), and these collectively reduced the estimated total number of admissions nationally by 2144 to 211,655 (a reduction of 1.0%). Thus, in the extremely unlikely event that every discrepancy in numbers of admissions was actually due to the opposite error to that we assumed, the estimated total number of admissions nationally would have been 213,799 (2144 or 1.0% greater).

At state/territory and national levels, the definition of an admission included only dogs brought in by the general public, and stray dogs that had been captured and not dogs transferred in. At that level, a transfer was considered to be an interim step in the dog’s admission. It was reasonable to assume that transfers in a particular state or territory went to other organizations in the same state or territory. Thus, to avoid counting a dog entering a pound then transferred to another organization as 2 admissions, for individual states and territories, we calculated intake as the sum of the intakes for all individual organizations in the state or territory minus the total number of transfers. Any particular dog could contribute multiple admissions in the same year.

For each of municipal councils pooled, individual animal welfare organizations, and rescue groups pooled, numbers of dogs that were reclaimed, rehomed, transferred and euthanized were expressed as percentages of the number of admissions where the outcome was known (i.e., the sum of numbers reclaimed, rehomed, transferred, other outcome and euthanized). Percentages ending in live release were calculated as the sums of reclaimed, rehomed and transferred percentages. Percentages rehomed and euthanized were also calculated for unclaimed admissions (impounded stray dogs not reclaimed by the owner). These allowed comparisons of rehoming performance between organizations with different proportions of admissions that were reclaimed (largely due to differing intake of strays versus owner surrenders). At state/territory and national levels, percentages were calculated disregarding transfers. For each state and territory, numbers of admissions where the dog was reclaimed, rehomed, experienced an “other” outcome, or was euthanized were calculated as the estimated total number of admissions multiplied by the respective percentages. National numbers were calculated as the sum of each state’s and territory’s numbers. 

### 2.8. Relationships between Council Attributes and Intakes and Outcomes

Associations between annual council intakes per 1000 residents and both council urban status (city or other) and resident population were assessed using councils with known intakes and resident populations of at least 5000 using linear regression. Associations between council outcomes (annual numbers reclaimed, rehomed, transferred out, and euthanized) per 1000 residents were each also assessed in the same way. Municipal council classifications of city (including rural city) or other were obtained from the Australian Bureau of Statistics [24]. Council intake per 1000 residents was simultaneously regressed on council urban status (city or other) and resident population using the -regress- command in Stata (version 14, StataCorp, Texas, TX, USA).

## 3. Results

### 3.1. Availability of Data and Contributions of Imputed Data

**Figure A2:**
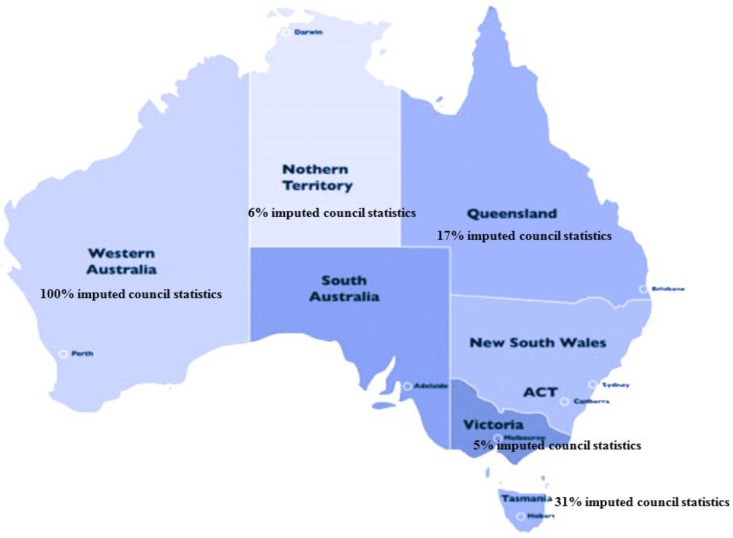
Map of Australia with imputed council statistics (proportions of the estimated numbers of admissions for the state or territory that were imputed municipal council admissions) in each state. No municipal council data were imputed for New South Wales, Australian Capital Territory or South Australia.

There were 515 possible sources of data: 496 municipal councils (excluding those identified as not having pounds or whose pounds were run by other organizations), and 19 animal welfare organizations thought to have ≥1000 dog admissions per year. Of these 515, we obtained data from 307 (60%), comprising 289 (58%) of the 496 municipal councils and 18 (95%) of the 19 welfare organizations (Table A1). The proportions of the estimated numbers of admissions for the state or territory that were imputed municipal council admissions were Victoria (5%), Northern Territory (6%), Queensland (17%), Tasmania (31%) and Western Australia (100%) (Figure A2). No municipal council data were imputed for New South Wales, Australian Capital Territory or South Australia. Excluding councils known to not have pounds, for Victoria, Northern Territory, Queensland, and Tasmania pooled, imputed councils were mostly non-city councils (2/61 or 3% being cities compared with 15/66 or 23% of non-imputed councils), and within each of these, the estimated resident populations were smaller for imputed councils (mean populations for imputed and non-imputed city councils: 70,154 and 98,707, respectively; and for imputed and non-imputed non-city councils: 17,433 and 30,144, respectively). Of Western Australia’s 138 councils, 18% (25) were cities and mean populations were 73,223 for city councils and 5375 for non-city councils. Annual council intakes per 1000 residents were not markedly affected by urban status of the council (i.e., city or non-city) with mean intake for city councils being only 0.3 admissions per 1000 residents less after adjusting for resident population) and council resident population also had little effect, with intakes declining by, on average, only 0.27 admissions per 1000 residents for each additional 10,000 residents in the council after accounting for council urban status. Similarly, annual council numbers reclaimed, rehomed, transferred out, and euthanized per 1000 residents were not markedly affected by either urban status of the council or council resident population. Mean numbers reclaimed, rehomed, transferred out, and euthanized per 1000 residents for city councils were, respectively, 0.8 more, 0.4 less, 0.3 more, and 0.3 more than for non-city councils. For each additional 10,000 residents in the council, annual council numbers per 1000 residents for these outcomes changed, respectively, by −0.17, −0.01, +0.07 and −0.07. These results indicate that accuracy of imputed council values would not have been markedly increased if imputations had been based on council urban statuses and resident populations.

Of the welfare organizations, only the RSPCA and Lost Dogs Home had comprehensive, publically available data at the state/territory and national levels. The Animal Welfare League provided data upon request for Queensland and South Australia, while their data for New South Wales was available online. Most of the smaller organizations (those thought to have intakes of ≥1000 to <5000 dog admissions per year) provided data on request for the study. The proportions of total dog admissions for the state or territory that were imputed for animal rescue group admissions varied by state/territory from 9% to 13%. For New South Wales, the estimated number of animal rescue group admissions was 15% of the state’s admissions.

### 3.2. National Results

Results nationally, by state/territory, and by organization type are shown in Table 1. Nationally, the number of dog admissions to municipal councils, animal welfare organizations and animal rescue groups in 2012–2013 was 211,655 or 9.3 admissions/1000 residents (Table 1). Of these, an estimated 48% or 101,037 (4.4/1000 residents) were reclaimed, 31% or 66,443 (2.9/1000 residents) were rehomed, and 21% or 43,900 (1.9/1000 residents) were euthanized. Of the 110,618 unclaimed admissions, an estimated 60% or 66,443 were rehomed, and 40% or 43,900 were euthanized.

Intakes into municipal councils, animal welfare organizations and animal rescue groups summed to 233,917. Of these, municipal councils accounted for 54% (125,149), animal welfare organizations accounted for 35% (81,929) and animal rescue groups 11% (26,839) (Table 1). The largest animal welfare organization was the RSPCA with an intake of 47,810 or 20% of the sum of the intakes. Percentage reclaimed was lower for welfare organizations (44% of intake) than councils (52% of intake), and animal rescue groups were assumed to have no admissions reclaimed by the original owners. 

Data were most complete for New South Wales, and based on our assumptions, animal rescue groups rehomed the highest proportions (98% of intake; 98% of unclaimed admissions). Animal welfare organizations rehomed 31% of intake (56% of unclaimed admissions), while councils rehomed 12% of intake (26% of unclaimed admissions). Thus, the proportion rehomed for councils pooled was about half that of welfare organizations for both proportions of total intake and of unclaimed admissions. In general, municipal councils transferred a greater proportion (31% of unclaimed admissions) than the welfare organizations (4% of unclaimed admissions) to other organizations, while the rescue groups were assumed not to transfer admissions. The proportion of intake that was euthanized for animal rescue groups was 2%, while councils and welfare organizations reported similar proportions of intake that were euthanized (20% and 22% of intake, respectively; 43% and 40% of unclaimed admissions, respectively).

### 3.3. Comparisons between States

New South Wales had the highest total number of admissions but the lowest on a per capita basis (56,252 or 7.7/1000 population) and Northern Territory the reverse (3095 or 13.1/1000 population; Table 1). Numbers reclaimed per 1000 residents varied from 7.9 (61% of admissions) in Northern Territory to 2.9 per 1000 population (37% of admissions) in New South Wales. Numbers rehomed per 1000 residents ranged from 3.8/1000 residents in Tasmania (36% of intake, 75% of unclaimed admissions) to 2.4/1000 residents in South Australia (26% of admissions, 55% of unclaimed admissions). Numbers euthanized per 1000 residents was lowest in Australian Capital Territory at 1.0/1000 residents (11% of intake, 23% of unclaimed admissions) and highest in Northern Territory at 2.5/1000 residents (19% of intake, 49% of unclaimed admissions). However, the proportions that were euthanized were greatest for New South Wales (29% of intake; 46% of unclaimed admissions).

### 3.4. Comparisons within Organizations between States

#### 3.4.1. Municipal Councils

Among municipal councils, those in Tasmania had the highest proportion reclaimed (79% of intake) while those in New South Wales had the lowest at 43%. Australian Capital Territory rehomed the greatest proportion of intake (30% of intake; 64% of unclaimed admissions). Tasmanian councils had the lowest proportions euthanized (4% of intake; 22% of unclaimed admissions) and New South Wales the highest (26% of intake; 46% of unclaimed admissions). 

#### 3.4.2. RSPCA

Within the RSPCA, intake was highest in Queensland (16,150) and lowest in Northern Territory (496). The proportions that were reclaimed varied from 61% in Australian Capital Territory to 12% in New South Wales, which at the time, was the only state RSPCA that did not include data for council pounds they managed. The proportion of intake that was rehomed ranged from 56% (70% of unclaimed admissions) in Northern Territory, to 22% (57% of unclaimed admissions) in Australian Capital Territory. Proportions that were euthanized were highest for RSPCA in New South Wales (39% of intake; 44% of unclaimed admissions) and lowest for RSPCA in Australian Capital Territory (8% of intake; 19% of unclaimed admissions). 

#### 3.4.3. Other Welfare Organizations

Within all states and territories, other welfare organizations rehomed higher proportions of intake than municipal councils, with the exception of Lost Dog’s Home in Queensland (9%; the same as for councils) (Table 1). Percentages rehomed of unclaimed dogs were highest for the AWL New South Wales (84%) followed by Dogs Home of Tasmania (80%) and AWL Queensland (60%). Based on our assumptions for animal rescue groups, they rehomed 12% of all admissions nationally, and of all dogs rehomed through pounds, shelters and animal rescue groups in Australia, rescue groups rehomed 39%. 

Lost Dogs Home in Victoria and Queensland euthanized higher proportions of their intakes and unclaimed admissions than councils in their corresponding states (18%, or 48% of unclaimed admissions in Victoria, and 20%, or 67% of unclaimed admissions in Queensland). For comparison, in Queensland, AWL euthanized 21% of intake (36% of unclaimed admissions) and the RSPCA 24% of intake (37% of unclaimed admissions) (Table 1). 

### 3.5. Trends over Time

In New South Wales, intake data from 2009–2012 [21] were available for municipal councils and the RSPCA (2009–2012) [15] and AWL [20,26]. Numbers of dogs admitted and euthanized declined substantially over time for RSPCA and AWL, and more modestly for municipal councils (Table 2). For RSPCA nationally, numbers of admissions and euthanasias also declined over these years [15]. 

## 4. Discussion

### 4.1. Admissions Nationally

Our study estimated the number of dog admissions to Australian municipal council pounds, animal welfare organization shelters and animal rescue groups in 2012–2013 and described their subsequent outcomes, based on the best available data from each state, and estimating and imputing where necessary. The estimated national number of admission of dogs into shelters was 211,655 in 2012–2013. Australia’s annual admission rate of 9.3/1000 residents was 75% of the United States of America’s admission rate in 2012–2013 of 12.5 /1000 residents (3.9 million dogs) [3]. When only admissions to welfare agencies were considered, the Australian rate of 3.6 dogs/1000 residents (81,929 dogs) was about 2.6 times that of the United Kingdom (1.4 dogs/1000 residents or 89,571 dogs in 2010) [4,5].

### 4.2. Admissions by State

Admission rates varied from 7.7 dogs (New South Wales) to 13.1 dogs/1000 residents in Northern Territory. Lower admission numbers are highly desirable. In United States of America (USA), a close correlation was observed between the numbers of dogs and cats admitted and numbers euthanized, and reduced intakes accounted almost entirely for the decrease in number of animals euthanized in shelters and municipal facilities between 1970 and 1995 [27]. There are also substantial costs to municipalities and welfare agencies for the admission and care of dogs until they are reclaimed, rehomed or euthanized. For example, the AWL in South Australia estimated the cost of shelter care to be $245/dog/per week, and when additional costs of preventive and veterinary care were included, the average cost to rehome a dog after one week of care was $1,056 [28]. Given that strategies aimed at decreasing numbers of admissions may be more effective at reducing costs and numbers euthanized than strategies that focus on rehoming animals, increasing resourcing to reduce admission numbers may be the more effective investment. 

Based on dog intake by state/territory, Northern Territory had the highest admission numbers, and although we could not determine the proportion of intake that was strays, the high percentage reclaimed (61%) indicates that the majority was strays and the owners were successfully identified. This suggests the high intake was partly due to poor dog containment measures by owners. Northern Territory has a significant number of indigenous communities (30% of Northern Territory population compared with 13% of national population) [29], which have culturally different attitudes to keeping and maintaining dogs, which might impact upon their wandering behavior [30,31]. However, the high percentage reclaimed indicates that people in this state generally care for and are generally responsible for their dogs [30].

New South Wales had the lowest admission rate (7.7/1000 residents), half that of Northern Territory and lower than Victoria (9.7/1000 residents). In Victoria, under state legislation, dogs can only be directly returned to the owner without impoundment if the dog is microchipped and has a current council registration (dog license) [32]. However, a proportion of impounded dogs are not reclaimed because owners cannot afford the impoundment fees [33]. Failure to reclaim an animal because of the owner’s inability or unwillingness to pay the impoundment costs, subsequently results in costs to the municipality or shelter to rehome or euthanize the dog. In addition, impoundment fees are typically not recovered when the dog is not reclaimed. Therefore, it is recommended that dogs without current council registration are directly returned where the owner can be identified, and the municipality seeks to recover some of these costs, while avoiding the more substantial costs potentially associated with an unclaimed dog. 

### 4.3. Admissions by Agency

Municipal council intakes accounted for the highest proportion of national admissions, but the economic burden to communities for managing stray and surrendered dogs is higher than represented by the 59% of intake to municipalities, because many outsource for a contracted fee, some or all dog intake to welfare agencies [33]. The estimated municipal cost of impoundment, rehoming and euthanasia of dogs ranges between $250 per admitted dog to in excess of $1000 [34,35], and this cost may increase when external pound service providers are used [36]. Therefore, it behooves municipal councils to focus more on decreasing intake, given this reduces both operational costs and euthanasia.

Animal welfare agencies accounted for 38% of national admission rate, and rescue groups 12%, and these organizations are largely funded by public donations [37,38], although the former also derive income from government contracts [32,39,40,41]. In Australia, the annual influx of pets into shelters incurs approximately $260 million in public and private funding annually, and savings from reduced dog admissions could be redirected to other programs [9]. 

### 4.4. Strategies to Reduce Intake

Low cost and free desexing programs targeted to locations and breeds contributing to high intake have been a major focus in the USA, and although conflicting results are reported, there is some evidence these programs decrease intake and numbers euthanized [27,37,42,43,44,45,46,47,48]. These strategies reduce both owner-surrendered [49] and stray (i.e., owned dogs that became lost) intake [50]. In Australia in 2013, approximately 78% of owned dogs were desexed [51], but only 15 to 22% of dogs being admitted to shelters were desexed, indicating an urgent need for investment in Australian programs targeted to locations contributing most to shelter and pound intake [52,53]. Other strategies aimed at reducing numbers of admissions of owned dogs are somewhat different from those that reduce stray dog admissions. A limitation of our data was that municipalities did not generally report the proportions of intake that were stray versus owner-surrendered. It is strongly recommended that future data collection includes this differentiation, and also reports outcomes for these subcategories.

Strategies which reduce stray dog admission include increasing the proportion of dogs identified with a tag and microchip to facilitate returning identified stray dogs to their owners without impounding [42,54], and assisting people with recurrent stray dog impoundments with fence repairs [55]. Although not widespread, in USA, some Animal Control Officers microchip in the field to increase the number of dogs identified with a microchip (pers comm K. Peterson, Salt Lake County Animal Services, USA). However, restrictive legislation in some states of Australia requires implanters to have extensive animal care and handling qualifications, which limits the number of animal control officers certified to microchip. 

Strategies to reduce surrender of owned dogs are based on research that shows most people surrendering their pet would keep it if the issues leading to surrender could be overcome [56]. Programs that assist people to keep their pet result in reduced numbers of animals euthanized and improved live release rates [27,37,42,57,58,59,60,61]. These programs include free behavior counseling services [62], assistance with health care costs, temporary boarding of pets during times of personal crises, food banks, training classes [63,64], and public education campaigns [1,62]. Strategies that reduce owner-surrendered admissions also include free or low cost desexing programs targeted to locations and breeds contributing to high intake.

In Australia and USA, the lack of rental accommodation allowing pets, or highly restrictive rental policies governing breed and size of dog, is one of the most common reasons people surrender animals, and accounts for 20 to 28%, and 29% of dog surrenders in Australia and USA, respectively [3,49,63,65]. In Australia, although 31% of the population lived in rented accommodation within the census periods of 2010–2011 and 2013–2014 [66], only 4% of advertised rentals allowed pets [67]. Pet owners stay twice as long, pay more rent and are no more likely to cause damage than non-pet owners, although renters with children cause an average of $150 more damage per unit per year [68]. Despite this, many landlords have a preconceived notion that pet owners cause more property damage [68]. Other advantages to landlords with a “Pets are Welcome” policy include shorter vacancy periods, lower vacancy rates, less advertising costs and more applicants per property [68]. 

Pet ownership results in health cost savings to the community. For example, total savings were estimated at $1.8 billion or 5% of Australia’s total health expenditure in 1999 [69]. It was reported that pet owners have reduced doctors’ visits and reduced use of medication for high blood pressure, high cholesterol, sleeping difficulties, and heart problems [69]. Given the substantial costs to municipalities and welfare agents for managing surrendered dogs, and the loss of individual and community health benefits of pet ownership, governments should legislate against unreasonable “no pets” clauses, in the same way it is illegal to discriminate against tenants with children. If legislating against unreasonable “no pets” clauses is unsuccessful, an alternative is taxing rental properties that do not allow pets. This could cover the costs for managing surrendered pets by shelters and pounds, and the loss of community health benefits, much the way that taxation is used on cigarettes to raise funds for medical costs subsequently incurred by smokers. This would provide an incentive for landlords to allow pets, and might be less contentious than mandatory anti-discrimination laws, but would be more difficult to enforce. 

### 4.5. Reclaim Percentage Nationally

The national percentage reclaimed of 48% compares favorably with USA’s reports, estimated at 15% to 30% of dogs admitted to shelters [70,71], and is close to the United Kingdom’s 2014 pound reclaim percentage of 54% [72,73]. Key factors influencing the probability of a dog being reclaimed are the proportion of owned dogs with some form of identification, such as microchip, collar and tag with owner contact details, or a council registration tag [73,74,75,76,77]. However, the accuracy of the contact information is also important. A 2015 study found 37% of stray dogs admitted to RSPCA Queensland had inaccurate microchip data, including the chip being registered to a previous owner or organization (47%), all phone numbers disconnected or incorrect (29%) or not registered with a database (14%) [75]. The latter is illegal and the implanter must register the chip within 7 days of implantation in Queensland [78]. Strategies that increase the proportion of dogs with correct owner contact details are needed, such as email reminders from database companies, and “Check a Chip” events where owners can bring their pet for scanning and updating information. 

### 4.6. Reclaim Percentages by State/Territory

The highest reclaim percentage was for Northern Territory (61%) and lowest for New South Wales (37%). Previous studies have directly linked socio-economic status with higher reclaim percentages and lower relinquishment numbers, both of which affect euthanasia percentages in shelters [74,79]. Of the Australian states/territories, Australian Capital Territory has the highest socio-economic status [80], but a lower reclaim percentage (53%) than Northern Territory, so clearly other factors are involved [81]. This is an area that warrants further research. Notably, New South Wales had the poorest performance in reclaimed dogs of all states both through pounds (43% reclaimed) and welfare organizations (12%). This was unexpected because New South Wales was the second state in Australia to mandate compulsory microchipping for dogs (Victoria 1998, New South Wales 2000). In contrast to other states, New South Wales provides lifetime registration for dogs [82]. However, owners are responsible for updating their contact details with the local council [83], and it is possible that over time, the lack of active updating by owners has made it difficult to link owners and dogs. 

Microchipped dogs had 2.5 times higher chance of being reclaimed compared to overall reclaim percentages for the shelters in a study in USA [76], and in a recent Australian study, microchipped dogs with no data problems had almost twice the chance of being reclaimed than non-microchipped dogs [75]. Strategies to increase the proportion of pets with identification are likely cost-effective if targeted to locations contributing disproportionately to shelter and pound intake of stray dogs, and include free or low cost microchipping, “Check a Chip” events [84], and free initial registration for dogs that are unregistered [85]. Offering a free engraved tag with owner’s contact details would facilitate return of lost dogs by neighbors, and is recommended to be included in microchipping events to reduce municipal costs associated with managing stray dogs. Utilization of web-based and social media platforms to locate owners also increases numbers of dogs reclaimed [86,87,88,89]. 

### 4.7. Reclaim by Agency

The national reclaim percentage for municipal councils (52%) was higher than welfare organizations (44%) which is expected because typically a higher proportion of their intake is strays rather than owner surrendered dogs. However, welfare organizations in Victoria achieve reclaim percentages equal to, or better than councils, which could reflect partially the number of council contracts held by welfare agencies, and that Victoria was the first state to mandate compulsory microchipping, and has a higher proportion of dogs microchipped relative to other states (pers comm Rick Walduck, Central Animal Records).

### 4.8. Rehoming—National

The national rehoming percentage was 31% of intake, and the proportion of unclaimed dogs rehomed was 60%. This percentage of intake rehomed of 31% is comparable to the dog rehoming statistics in USA (estimated at 35% annually [3]). There is a positive correlation between training of shelter dogs and rehomeability [64,65,90,91], and supporting new owners of adopted dogs to manage behaviors through training classes and counseling is integral to the success of the rehoming process [92,93]. 

### 4.9. Rehoming—State and Agency Comparisons

Both Tasmania and Australian Capital Territory had the highest rehoming percentages at 36% (5% above the national rehoming percentage). National rehoming percentages for municipal councils (12% of intake and 26% of unclaimed dogs) were less than half that of welfare agencies (31% of intake and 56% of unclaimed dogs). Although some councils do offer rehoming services, transfer rates were much higher for councils than welfare agencies, because, rather than rehoming unclaimed dogs, many councils transfer unclaimed dogs to welfare organizations and rescue groups. 

Strategies that increase rehoming success include innovative marketing of dogs for adoption, and utilizing rescue and foster network systems [94,95]. Foster and rescue networks are invaluable because they reduce facility overcrowding, thereby reducing disease and behavioral issues [95]. They also provide behavior conditioning in a foster care environment [95,96] and extend the reach of the adoption program to potential adopters beyond the vicinity of the shelters [95,97]. It is recommended that councils increase utilization of fostering and rescue groups in the community, and implement more innovative marketing strategies to increase adoptions.

### 4.10. Transfer—National, State and Agency Comparisons

Municipal councils transferred more dogs (32% of their unclaimed admissions) to other organizations than other types of agencies (4% of their unclaimed admissions). This in part reflects contractual arrangements that exist where unclaimed dogs are transferred after the mandatory holding period to a welfare agency (council-mandated euthanasias excepted). Some of these organizations then transfer dogs to rescue groups who may specialize in certain breeds, or be able to offer long-term foster placement to deal with physical or behavioral issues [98]. These strategies serve to reduce numbers euthanized and should be utilized where possible by municipal and welfare agencies. 

### 4.11. Euthanasia—National and State Comparisons

In 2012–2013, 43,900 dogs (1.9 /1000 residents) were euthanized, which represented 21% of national admissions or 40% of unclaimed admissions. This compared favorably to recent euthanasia estimates for USA at 1.2 million dogs (3.7 dogs/1000 residents in 2016) [3] or 31% of admissions [3], although the USA’s euthanasia statistics did not include data from foster-based rescue groups, where euthanasia percentages are normally lower. Australia’s euthanasia percentage compares poorly against the United Kingdom’s euthanasia percentage, estimated to be 10% of admissions to animal welfare organizations in 2010 [5], and 6% of admissions to municipal councils in 2010–2011 and 2014 [7,99]. Euthanasia percentage reflects efficacy of programs aimed at reducing intake and increasing the numbers of dogs reclaimed, rehomed, transferred and fostered. The less effective these processes are, the higher the number of dogs euthanized. Clearly, some states were more effective than others at these processes. New South Wales had the highest euthanasia percentage at 29% of admissions (8% above national average) with 46% of unclaimed admissions ending with the dog being euthanized. In contrast, all the other states had euthanasia percentages between 11% of admissions (Australian Capital Territory) and 21% of admissions (Western Australia and South Australia). The estimated national number of admissions that ended in live release in Australia was 167,480 dogs (7.3 /1000 residents) or 79% of admissions. The goal of ≥90% live release percentage or <10% euthanasia percentage is generally accepted as representing zero euthanasia of healthy and treatable animals [100]. Based on data on municipal websites, 25% of Victorian pounds achieved live release percentages of 93% or higher for dogs. 

### 4.12. Euthanasia—Agency Comparisons

Municipal councils (20% of admissions) and welfare agencies (22% of admissions) had comparable euthanasia percentages in 2012–2013. Welfare agencies performed slightly better when the proportions of unclaimed dogs that were euthanized were compared (43% versus 40%). Municipal councils are responsible for mandated euthanasia of dangerous dogs and banned breeds, although this is also included in contractual agreements between councils and welfare agencies (pers communication, David Peters, CEO Dogs’ Home of Tasmania, 2014, Tasmania).

### 4.13. Data Quality

Our study identified the substantial difficulties in obtaining comprehensive and meaningful shelter and pound data in Australia. There was no existing centralized national database where municipal council pound and welfare agency statistics were collated. At the state level, only New South Wales had comprehensive centralized data [21] but at the time of writing in February 2017, it had not been updated since 2011–2012. In South Australia, the Dog and Cat Management Board data did not include numbers of dogs rehomed or euthanized for councils, limiting the usefulness of the data. Only in Victoria were individual municipal data publically available to municipal taxpayers, albeit for only 73/79 councils at the time of the study, and some were out of date. Of welfare agencies, only the RSPCA and LDH were fully transparent with current data on their websites. For most other welfare agencies, the information was not accessible to the public, but was provided on request for this study. Municipal data could have been requested under the “Right to information” legislation, but this strategy was not used because of the number and cost of applications required (for example, $37 per application in Tasmania and $44.85 per application in Queensland in 2015) and the lengthy timeframe to gain access to the data. Moreover, application of this Act is limited to municipal councils and does not apply to welfare agencies. Council pounds are funded by public taxes/rates, and taxpayers are therefore entitled to know how their money is being spent. Although there is no legal requirement for welfare organizations to publish their shelter data, there is a moral obligation as these organizations are funded by donations from the animal-loving public, and it could logically be expected that they should report on the allocation of their resources to their donors. Publishing data can provide a strong impetus for change and there have been numerous anecdotal reports in Australia and from overseas of substantially improved outcomes for animals in response to public pressure [101,102,103,104], and this warrants further research. In USA, shelter data has driven charitable fund allocation to effective and evidence-based shelter/pound programs [18,105,106]. 

These data quality limitations could all be overcome with careful planning and implementation of an appropriate data collections system mandated at the state level under domestic animal management legislation. Collated and individual agency data should be publicly available at the state level for municipal councils, welfare agencies and rescue groups, with state/territory figures adjusted to account for double counting of admissions where the dog is transferred. It is recommended that all municipal councils in Australia be required to have a Domestic Animal Management Plan similar to that mandated in Victoria which outlines the services, programs and policies the Council has established to address management of dog and cat issues in their community [22] and reports annually on the statistics of each species of animals admitted and their outcomes [22]. Differences in sources and attributes of surrendered and stray dogs may partly account for differences in reclaim percentages by state/territory, and indeed in rehome and live release percentages. As strategies differ for decreasing intake and increasing proportion of reclaimed and rehomed, reporting the subcategories that end in live release is recommended. Because effective strategies to reduce intake will depend on the source and age of dogs, it is recommended that admission data includes whether the dog is owner-surrendered or stray, age, and also microchip and desex status to guide management programs. Agencies receiving transferred dogs should be noted in data collected. Data should include numbers in care at the end of the reporting period and also numbers in foster care, because the latter is an effective strategy to increase numbers rehomed [107,108]. Greater detail of subcategories of intake and outcome data would facilitate more meaningful comparisons between states and agencies. 

National standardization of data categories is also essential, and is not necessarily clear even within organizations. In our experience, most welfare agencies do not report in their intake or euthanasia statistics, dogs surrendered with a request for euthanasia. These animals may be redirected to the shelter veterinary clinic or external veterinarian, but account for a significant proportion of dogs euthanized. For example in RSPCA in Queensland, although such admissions constituted only a small proportion of intake in the study year (6% or 908 dogs), they represented 23% of dogs euthanized (pers comm, M. Patterson, RSPCA, Queensland). This reporting is further complicated because owners requesting euthanasia may also be referred to the shelters by private veterinary clinics [27]. It is recommended that dogs surrendered for euthanasia are reported, but in a separate category.

### 4.14. Contribution of Rescue Groups

Rescue groups had the highest assumed rehoming percentages of all organizations, which was expected based on data from the largest state in Australia (NSW) where only 2% were euthanized. The number of dogs reclaimed by rescue groups was assumed to be insignificant given that rescue groups cannot legally accept strays. The majority of dog admissions to rescue groups are from welfare agencies and municipal pounds and transfer only occurs after the legally mandated minimal holding period. The bulk of the remaining admissions to rescue groups are by legal surrender direct from owners, which has substantially zero reclaim rates. Although responsible for only 12% of national admissions, they accounted for an estimated 39% of national adoptions, offering a very effective pathway to improving live release rates and reducing euthanasia [105]. However, it is likely that being volunteer-dependent, there is a limit to the numbers of dogs such organizations can manage. 

### 4.15. Limitations of Study

There were a number of limitations of the study, which have been summarized in Appendix Table A2, and these may have resulted in over- or under-estimation of intakes, and numbers and percentages of intakes ending in the various outcomes including euthanasia. In Western Australia all the council data were imputed because data were obtained for only 9/140 councils. However, the state represented only 11% of the Australian population, minimizing the impact of imputation errors on overall results. Councils with imputed data were less often classified as cities compared with those with actual data, and within each of city and non-city councils, councils with imputed data had smaller resident populations. However, neither council urban status (city or non-city) nor council resident population substantially affected intakes or numbers reclaimed, rehomed, transferred out, and euthanized per 1000 residents. While we assumed rescue groups in other states performed similarly to NSW, to exclude rescue groups from the study would have been a more serious omission, given their substantial contribution to rehoming of dogs. We assumed that data were the same for year to year for municipal councils with data only available for years other than 2012–2013. However, this is unlikely to be true, given the general improvement evident in NSW. Therefore, this assumption would have overestimated euthanasia, but their contribution to the overall figures was relatively minor. Exclusion of smaller organizations will have led to an underestimation of intake and rehoming, but numbers euthanized would be less affected given many of these small welfare groups have high live release rates. 

There were also substantial limitations of data quality. Definitions of eligibility for inclusion were unclear, detailed data describing sources of dogs transferred in, and destinations for dogs transferred out, were not available, annual numbers did not always reconcile, numbers in care at the start and end of the year were often not available (with in-care numbers instead added to admissions for RSPCA), and it was difficult to ascertain whether some data for municipal council pounds run by animal welfare organizations were included in municipal council data, animal welfare organization data, both or neither. However, the estimates in this paper are the best available currently for Australia and highlight the need for transparency and standardization of data, particularly among municipal councils, but also for some major and most smaller welfare organizations. 

## 5. Conclusions

It is recommended that a strategy of state-based reporting of pound and shelter statistics in a standardized and epidemiologically sound manner be adopted, and the results made publicly available, incorporating rescue organizations where possible. Once such data are available, a detailed investigation of how management strategies impact on dog admissions and outcomes, especially live release and euthanasia, is warranted. The findings could be used to identify low-performance areas and to guide the development of targeted surveillance and interventions. Results from our study provide a starting point for Australia to benchmark its dog management policies and performance against comparable countries, and to re-evaluate existing strategies to improve the efficiency of managing stray and surrendered dogs.

## Figures and Tables

**Table 1:** Council pound and shelter statistics by state.

State/Territory & Organization	No. Admissions (Intake)	No. Reclaimed	No. Rehomed	No. with Other Outcome ^1^	No. Transferred	No. Euthanized	In-care Population (Start of yr)	In-care Population (End of yr)	Assumed Overstated Intake ^2^	Assumed Understated Intake ^3^	% Reclaimed	% Rehomed	% Rehomed (of Unclaimed)	% live Release ^4^	% Euthanized	% Euthanized (of Unclaimed)
*NEW SOUTH WALES* (human population 7,307,183)																
Municipal councils pooled	46,486	19,867	4781	0	9279	11,945			614	0	43%	10%	18%	74%	26%	46%
RSPCA	10,438	1208	4693	45	452	4021	633	652	0	0	12%	45%	51%	61%	39%	44%
AWL	1074	0	904	0	31	139			0	0	0%	84%	84%	87%	13%	13%
Animal rescue groups pooled	8630	0	8457	0	0	173			0	0	0%	98%	98%	98%	2%	2%
No. admissions for state ^5^	56,252	21,082	18,842	45		16,283										
Number per 1000 human residents/% of admissions where outcome was known	7.7	2.9	2.6	0.0		2.2					37%	33%	54%	71%	29%	46%
*VICTORIA* (human population 5,632,521)																
Municipal councils pooled	18,720	11,505	3162	0	680	3155			469	250	62%	17%	45%	83%	17%	45%
RSPCA	12,882	7259	3636	43	11	1973	525	485	0	0	56%	28%	64%	84%	15%	35%
Animal Aid Victoria	3043	1983	724	0	0	317			19	0	66%	24%	70%	90%	10%	30%
Save a Dog	1061	964	486	0	0	14			0	403	66%	33%	97%	99%	1%	3%
Lost Dogs Homes	12,933	8253	2319	0	104	2279			0	22	64%	18%	49%	82%	18%	48%
Lort Smith Animal Hospital (AWL)	242	0	127	0	94	21			0	0	0%	52%	52%	91%	9%	9%
Animal rescue groups pooled	6652	0	6519	0	0	133			0	0	0%	98%	98%	98%	2%	2%
No. admissions for state ^5^	54,831	29,942	16,960	43		7886										
Number per 1000 human residents/% of admissions where outcome was known	9.7	5.3	3.0	0.0		1.4					55%	31%	68%	86%	14%	32%
*SOUTH AUSTRALIA* (human population 1,656,035)																
Municipal councils pooled	7723	5412	536	0	757	1018			0	0	70%	7%	23%	87%	13%	44%
RSPCA	3879	1800	1043	11	2	618			0	0	52%	30%	62%	82%	18%	37%
Animal Welfare League	5248	1998	1155	0	24	2064			7	0	38%	22%	36%	61%	39%	64%
Animal rescue groups pooled	1956	0	1917	0	0	39			0	0	0%	98%	98%	98%	2%	2%
No. admissions for state ^5^	14,833	7757	3917	9		3150										
Number per 1000 human residents/% of admissions where outcome was known	9.0	4.7	2.4	0.0		1.9					52%	26%	55%	79%	21%	45%
*AUSTRALIAN CAPITAL TERRITORY* (human population 375,200)																
Domestic Animal Services	1439	772	426	0	16	225			0	0	54%	30%	64%	84%	16%	34%
RSPCA	1730	1072	389	9	152	132	100	76	0	0	61%	22%	57%	92%	8%	19%
Animal rescue groups pooled	443	0	434	0	0	9			0	0	0%	98%	98%	98%	2%	2%
No. admissions for state ^5^	3444	1831	1241	9		363										
Number per 1000 human residents/% of admissions where outcome was known	9.2	4.9	3.3	0.0		1.0					53%	36%	77%	89%	11%	23%
*QUEENSLAND* (human population 4,568,205)																
Municipal councils pooled (excluding councils where RSPCA or other animal welfare organization runs pound)	26,278	14,657	2524	0	7412	4412			311	3038	51%	9%	18%	85%	15%	31%
Animal Welfare League QLD	5233	2169	1829	0	127	1095			24	0	42%	35%	60%	79%	21%	36%
Lost Dogs Home (Wirra & Willawong)	3612	2512	342	0	29	743			0	14	69%	9%	31%	80%	20%	67%
RSPCA (added 908 request euthanasia)	16,150	5568	5809	134	717	3907	746	761	0	0	35%	36%	55%	75%	24%	37%
Animal rescue groups pooled	5395	0	5287	0	0	108			0	0	0%	98%	98%	98%	2%	2%
No. admissions for state ^5^	51,100	24,908	15,792	134		10,266										
Number per 1000 human residents/% of admissions where outcome was known	11.2	5.5	3.5	0.0		2.2					49%	31%	60%	80%	20%	39%
*NORTHERN TERRITORY* (human population 235,881)																
Municipal councils pooled	3139	1753	92	0	550	475			283	15	61%	3%	8%	83%	17%	43%
RSPCA	496	94	253	0	0	106	50	93	0	0	21%	56%	70%	77%	23%	30%
Animal rescue groups pooled	279	0	273	0	0	6			0	0	0%	98%	98%	98%	2%	2%
No. admissions for state ^5^	3095	1873	627	0		595										
Number per 1000 human residents/% of admissions where outcome was known	13.1	7.9	2.7	0.0		2.5					61%	20%	51%	81%	19%	49%
*WESTERN AUSTRALIA* (human population 2,437,994)																
Municipal councils pooled	19,144	10,536	4052	29	0	4489										
RSPCA	713	303	254	0	0	163	101	94								
Animal rescue groups pooled	2879	0	2822	58	0	0										
No. admissions for state ^5^	22,707	10,839	7128	29		4710										
Number per 1000 human residents/% of admissions where outcome was known	9.3	4.4	2.9	0.0		1.9					48%	31%	60%	79%	21%	40%
*TASMANIA* (human population 512,106)																
Municipal councils pooled (other than those whose pounds were run by Dogs’ Home of Tasmania or RSPCA)	2251	1783	21	0	345	101			0		79%	1%	5%	96%	4%	22%
Dogs’ Home of Tasmania	1673	422	810	0	0	205			312		29%	56%	80%	86%	14%	20%
RSPCA	1522	652	550	5	0	343	102	74	0		42%	35%	61%	78%	22%	38%
Animal rescue groups pooled	605	0	593	0	0	12			0		0%	98%	98%	98%	2%	2%
No. admissions for state ^5^	5393	2803	1937	5		649										
Number per 1000 human residents/% of admissions where outcome was known	10.5	5.5	3.8	0.0		1.3					52%	36%	75%	88%	12%	25%
*NATIONAL* (human population 22,725,125)																
No. admissions ^5^	211,655	101,037	66,443	275		43,900										
Number per 1000 human residents/% of admissions where outcome was known	9.3	4.4	2.9	0.0		1.9					48%	31%	60%	79%	21%	40%
*AGENCIES NATIONALLY*																
All councils across states	125,149	66,286	15,594	29	19,040	25,820					52%	12%	26%	80%	20%	43%
RSPCA National	47,810	17,956	16,627	247	1334	11,263					38%	35%	56%	76%	24%	38%
Other welfare agencies combined	34,119	18,301	8696	0	409	6877					53%	25%	54%	80%	20%	43%
Animal rescue groups pooled	26,839	0	26,302	58	0	479					0%	98%	98%	98%	2%	2%
Combined RSPCA & welfare orgs											44%	31%	56%	77%	22%	40%

^1^ Other outcomes consisted of dogs that experienced an outcome other than being reclaimed, rehomed, transferred or euthanized, and included dogs that died in the shelter, were stolen or lost etc. ^2^ After accounting for any differences between the numbers in care at the start and end of the year, the intake number was more than the total of the numbers of admissions ending in each of the 5 possible outcomes. For these, we assumed numbers for each outcome and numbers in care were correct and intake numbers overstated, so deducted the excess from the state’s or territory’s recorded total intake. ^3^ After accounting for any differences between the numbers in care at the start and end of the year, the reported intake was less than the total of the numbers of admissions ending in each of the 5 possible outcomes. For these, we assumed numbers for each outcome and numbers in care were correct, and intake numbers understated, and added the discrepancy to the organization’s state’s or territory’s recorded total intake. ^4^ Percentage of admissions ending in reclaiming, rehoming or transfer. ^5^ Number of admissions for state or territory is less than the sum of each organization’s intake as transfers were deducted from the state or territory total intake. Numbers of admissions ending in each of the dog being reclaimed, rehomed, experiencing an other outcome and being euthanized for the state or territory were calculated as number of admissions for state or territory multiplied by the percentage of intake that ended in that outcome.

**Table 2:** Numbers of dog admissions to municipal councils, RSPCA and Animal Welfare League in New South Wales, and numbers euthanized from 2009–2010 to 2011–2012.

Organizations	Year	No. of Dog Admissions	% Decrease	No. of Dogs Euthanized	% Decrease
Municipal councils pooled	2009–2010	49,958		15,461	
	2010–2011	48,427	3.1	13,661	11.6
	2011–2012	46,486	4.0	11,945	12.6
% decrease over 2 years ^1^			6.9		22.7
RSPCA	2009–2010	21,328		8361	
	2010–2011	20,959	1.73	8209	1.8
	2011–2012	11,989	42.8	4862	40.8
% decrease over 2 years ^1^			43.8		41.8
Animal Welfare League	2009–2010	1882		887	
	2010–2011	1816	3.5	717	19.2
	2011–2012	1427	21.4	297	58.6
% decrease over 2 years ^1^			24.2		66.5

^1^ Reduction from 2009–2010 to 2011–2012 expressed as percentage of number in 2009–2010.

## Abbreviations

The following abbreviations are used in this manuscript:

RSPCA	Royal Society for the Prevention of Cruelty to Animals
LDH	Lost Dogs’ Home
AWL	Animal Welfare League
USA	The United States of America

## Appendix A

**Table A1:** Sources of data by state and territory (for 2012–2013 unless otherwise indicated).

State or Territory	Aggregated Data for State/Territory: Available (Yes/No) /Source	No. of Municipal Councils Participating/No. in State Excluding Those Known to Not Have or Run Pound Facilities	Source of Council Information	No. of Welfare Organizations (Thought to Have ≥1000 Dog Intake Per Year) Participating/No. in State	Participating Welfare Organizations Included	Welfare Organization’s Information Sourced from:	Other Included Rescue Groups in the State
Western Australia	No	9/140 (Numbers of municipal councils that did not have pound facilities or whose pounds were operated by another organization were not determined.)	Individual municipal councils by direct approach	1/1	RSPCA	Annual report online [15]	Rescue groups (imputed)
Northern Territory	No	5/8 (The remaining 8 municipal councils did not have pound facilities)	Individual municipal councils by direct approach	1/1	RSPCA	Annual report online [15] and additional detail by direct correspondence	Rescue groups (imputed)
South Australia	Yes/Collated council data	74/74 (As aggregated data were available, numbers of municipal councils that did not have pound facilities or whose pounds were operated by another organization were not determined.)	Dog and Cat Management Board	2/2	RSPCA, AWL	Annual report online (RSPCA) Direct correspondence (AWL)	Rescue groups (imputed)
Queensland	No	9/64 (6 of the remaining 14 municipal councils did not have pound facilities and 8 councils’ pounds were operated by another organization)	Individual municipal councils by direct approach	3/3	RSPCA, AWL, LDH	Annual report online/on request (RSPCA [15] ^1^, LDH) Direct correspondence (AWL)	Rescue groups (imputed)
New South Wales	Yes/annual reports	152/152 (As aggregated data were available, numbers of municipal councils that did not have pound facilities or whose pounds were operated by another organization were not determined.)	Department of the Premier and CabinetMost recent available-2011–2012 report [21] Max for Justice (transfer numbers to rescue groups)	3/3	RSPCA Lost Dogs Home (LDH) Animal Welfare League (AWL)	Annual reports available online (RSPCA [15], LDH [109]) AWL (—data from Discussion paper/May 2012/NSW Companion Animal taskforce [20]) Direct correspondence Personal communication	Max4JusticeRescue groups (imputed)
Australian Capital Territory	Yes/single pound operated by Domestic Animal Services	1/1	Domestic Animal Services	1/1	RSPCA	Annual report online [15] Direct correspondence—Domestic Animal Management (DAM)	Rescue groups (imputed)
Victoria	No	32/38 (The remaining 41 municipal councils’ pounds were operated by another organization)	Domestic Animal Management Plan on individual council websites (various years depending on council 2006 to 2013)	5/6	RSPCA, LDH, Animal Aid Victoria, Lort Smith Hospital and Save-a-Dog	Annual report online (RSPCA [15], LDH [109]) Direct correspondence (Save a Dog, Lort Smith)	Rescue groups (imputed)
Tasmania	No	7/19 (The remaining 10 municipal councils’ pounds were operated by RSPCA or Dogs’ Home of Tasmania)		2/2	RSPCA Dogs’ Home of Tasmania	Annual report online [15] Direct Correspondence (Dogs’ Home)	Rescue groups (imputed)

^1^ The number of owner-surrendered admissions where euthanasia was requested (*n* = 908) was added to reported numbers of both admissions and euthanasias to make Queensland RSPCA numbers comparable to those for the RSPCA in other states and territories.

**Table A2:** Limitations of the study: issues resulting in over/under reporting of data.

Over-Reporting of Numbers	Under-Reporting of Numbers
**Potential double counting where inability to separate pound and shelter data exists (welfare agencies contracted to run council pounds).** e.g., Launceston pound is operated by RSPCA and data were probably included in shelter statistics. LDH runs pound services for multiple councils in Queensland, where pound data is included in shelter statistics.	**Unavailable data/lack of transparency from municipal councils and pounds through non-responses, delayed responses, unavailable data categories.** e.g., Justice for Max was unable to separate the rescue data that have been submitted by the rescue groups by species, resulting in an estimation of the number of dog admissions by the rescue organizations. e.g., lack of response from large numbers of councils in Western Australia, where only 9/140 councils provided data on request.
**Possible transfers of dogs across states/shelters within the same state.** e.g., Dogs’ Home of Tasmania transferred dogs among their 3 facilities, while others, including RSPCA and AWL, move animals across state borders. These transferred animals might be double counted in both states.	**Some municipal councils’ management practice of returning dogs that were reclaimed by owners by without being housed in the organization’s facilities. These dogs were most likely excluded in that organization’s intake numbers.**
**Some organizations collate data from multiple sites, such as transfers from different pounds, resulting in re-admission of same dogs.**	
**Discrepancy in the numbers, often associated with “other outcomes”, which can result in over or under-reporting of data.** e.g., dogs in foster care, differences in reporting periods (calendar versus financial), dogs that died/escaped and dogs already impounded before the initiation of the reporting period with a subsequent recorded outcome.	
**Extrapolation process to account for potential over/under reporting can introduce some variances.** e.g., the numbers of animals in care may have been either over- or under-stated. This has been accounted by for each state and territory (the sum of these assumed discrepancies was specifically listed in results and added or deducted from the total number of admissions), based on the relevant scenario described, and hence results in under/over reporting of data.	
**Other issues affecting the accuracy of reporting.** (i) Variations existed in the periods for which data were available. Some was reported based on calendar year and others on financial year. While the study utilized the most recent data available (usually 2011–2012 or 2012–2013), some information was older. In Victoria, pound data are available via the Domestic Animal Management Plan on individual council websites and were retrieved from years ranging from 2006 to 2013. This further affected the accuracy of the data collated. (ii) Although relatively few dogs (<1%) were involved, there were some discrepancies in the raw data often associated with “other outcomes” (such as foster care, escaped) and in the numbers of animals in care at the start and end of the reporting periods. Depending upon the direction of these variations, they could result in over or under-reporting. (iii) It was assumed that all shelters/pounds in a state would function in a similar way to others in that state i.e., that small rural shelters would function similar to those in major cities, in terms of admissions and outcomes of dog population in the shelters. It is unknown how accurate this assumption was, and can contribute to under or over estimation of numbers. (iv) Welfare organizations with admissions of less than 1000 dogs annually were not included, leading to some underestimation of state and national admissions. (v) Only data for rescue groups in NSW were available and was estimated to be 75% complete (pers comm Geoff Davidson, Justice4Max/New South Wales) but this was assumed to be representative of rescue groups contribution in other states, leading to errors which may under or over-estimate dogs rehomed. Contribution to intake was relatively small.	
